# Epigenetic induction of tumor stemness via the lipopolysaccharide-TET3-HOXB2 signaling axis in esophageal squamous cell carcinoma

**DOI:** 10.1186/s12964-020-0510-8

**Published:** 2020-02-03

**Authors:** Fengkai Xu, Zhonghe Liu, Ronghua Liu, Chunlai Lu, Lin Wang, Wei Mao, Qiaoliang Zhu, Huankai Shou, Kunpeng Zhang, Yin Li, Yiwei Chu, Jie Gu, Di Ge

**Affiliations:** 1 Department of Thoracic Surgery, Zhongshan Hospital, Fudan University, 180 Fenglin Road, Shanghai, 200032 People’s Republic of China; 2 Key Laboratory of Medical Epigenetics and Metabolism, Institute of Biomedical Sciences, Fudan University, Shanghai, People’s Republic of China; 3 Department of Immunology, Fudan University, Shanghai, People’s Republic of China

**Keywords:** Lipopolysaccharide, Stemness, Esophageal squamous cell carcinoma, Ten-eleven-translocation (TET), Homeobox B2 (HOXB2)

## Abstract

**Background:**

Esophageal squamous cell cancer (ESCC) is one kind of frequent digestive tumor. The inflammatory environment plays an important role in the tumorigenesis and development of ESCC. Cancer stem cells are a small group of tumor cells with stem cell characteristics, which can potentially hinder the tumor management and treatment.

**Methods:**

ELISA was performed to detect the lipopolysaccharide concentration in cancer tissues. qPCR, Western blot, FACS, Immunohistochemistry, Immunofluorescence and Dot blot were applied to detect target genes expression. CCK-8, Colony-formation, Transwell, Sphere and Xenograft were conducted to investigate the function of cells, influenced by risk factors. The survival curve was drawn with the Kaplan-Meier product limit estimator. Nano-hmC-Seal-seq was utilized to detect the downstream target of TET3. ChIP-qPCR was adopted to demonstrate the transcriptional regulation of stem cell-associated genes by HOXB2.

**Results:**

Lipopolysaccharide concentration was significantly up-regulated in ESCC. High concentration of lipopolysaccharide stimulation induced the stemness of ESCC cells. TET3 expression was elevated with lipopolysaccharide stimulation via p38/ERK-MAPK pathway in ESCC and negatively correlated with patients’ survival. TET3 induced the stemness of ESCC cells. Nano-hmC-Seal-seq showed that TET3 overexpression led to a significant increase in 5hmC levels of HOXB2 gene region, which was thus identified as the downstream target of TET3. The binding of HOXB2 to NANOG and cMYC was verified by ChIP-qPCR.

**Conclusions:**

Lipopolysaccharide served as a tumor promotor in ESCC by inducing cancer cell stemness through the activation of a LPS-TET3-HOXB2 signaling axis, which might provide a novel therapeutic strategy for ESCC.

Video Abstract

## Background

Esophageal squamous cell carcinoma (ESCC) is the main pathological type of esophageal cancer in China, which accounts for more than 90% [1]. ESCC is characteristic of high malignancy, latent early symptoms, and rapid metastasis, which is usually asymptomatic in a large number of patients until an advanced disease stage [2]. Although surgery, radiotherapy and chemotherapy are the current mainstay of treatment of ESCC, the prognosis remains poor due to high tendency of recurrence and metastasis. As a matter of fact, the worldwide 5-year survival rate of ESCC is no more than 20% [1]. Therefore, the deeper study in the molecular mechanism of the tumorigenesis and development of ESCC, which might contribute to more effective management and treatment, is of great clinical value.

The inflammatory environment plays an important role in the tumorigenesis and development of esophageal cancer [3]. Lipopolysaccharide (LPS) is one of the main components of the cell wall of Gram-negative bacilli [4]. Since Gram-negative bacteria are the main environmental pollutants, people’s daily life is unavoidable to be exposed to LPS [5]. The esophagus, a hollow viscera, is frequently stimulated by LPS along with eating behavior. LPS has been reported to promote the proliferation and migration of various tumors including ESCC [6–8]. However, no study has investigated whether LPS could regulate the stemness of ESCC.

Tumor stemness refers to an extremely tiny group of cancer cells with stem cell characteristics, which results in tumorigenesis, maintaining tumor growth and heterogeneity, promoting distant metastasis, and inducing therapeutic tolerance [9]. Thus, the cancer stem cells (CSCs) become a potential cause of failure to cancer treatment. For the reason that traditional treatment for ESCC is unsatisfactory currently [1], it might be a novel breakthrough to explore the molecular mechanism of the stemness of ESCC cells, which is expected to be the potential therapeutic target.

Epigenetic modification has been proved to regulate the multi-step process of tumor development [10, 11]. DNA methylation is one of the main forms of epigenetic modification, and various methylases have been reported to regulate the stemness of various tumors [12, 13]. DNA methylation levels are maintained by the dynamic balance between methylases and demethylases [14]. Ten-eleven-translocation (TET) methylcytosine dioxygenase family, a member of demethylases, catalyzes the stepwise oxidation of 5-methylcytosine (5mC) to 5-hydroxymethylcytosine (5hmC) in DNA, which exerts a demethylation function [15]. It has been reported that TET protein is involved in the regulation and maintenance of the stemness balance of mesenchymal stem cells and embryonic stem cells [16, 17]. In the field of cancer research, Cui and colleagues disclosed that down-regulation of TLX could induce TET3 expression and inhibit glioblastoma stem cells’ self-renewal and tumorigenesis [18]. However, no study has demonstrated the correlation between the TET family and the stemness of tumor cells in ESCC.

In the present study, we found that LPS could induce the stemness of ESCC cell through the up-regulation of TET3 expression. Furthermore, we identified Homeobox B2 (HOXB2) as the downstream target of TET3. In mechanism, we proved that LPS activated p38/ERK-MAPK signaling pathway to up-regulate TET3 expression. Then, HOXB2 was activated through the demethylation function of TET3 and promoted the transcription activity of stemness-related genes, cMYC and NANOG. These findings indicated that LPS functioned as a tumor promotor in ESCC by inducing cancer cell stemness through LPS-TET3-HOXB2 signaling axis, which might provide a novel therapeutic strategy for ESCC.

## Materials and methods

### Tissue microarray, tissue sample and immunohistochemistry

The tissue microarray enrolled 299 ESCC patients who underwent radical esophagectomy in the Department of Thoracic Surgery, Zhongshan Hospital, Fudan University, China, in 2007. Each patients’ information of follow-up was recorded in details. The follow-up was conducted as previous description [19]. Patients’ clinicopathological data were collected in details, including sex, age, tumor pathology, tumor size, tumor depth, lymph node metastasis, tumor-node-metastasis (TNM) stage (according to American Joint Committee on Cancer, eighth edition) [20], histological grade, and overall survival. The overall survival was defined as the interval from surgery to death. In addition, 10 pairs of fresh ESCC tissues and para-cancerous tissues were obtained from 10 ESCC patients at Zhongshan Hospital in 2017. The written informed consent was obtained from each patient. This study was approved by the ethics committee on human research of Zhongshan Hospital, Fudan University, and conducted according to the principles of the Declaration of Helsinki.

Immunohistochemistry (IHC) staining and evaluation were consistent to the previous research [21]. The antibody was listed in Additional file 1: Table S1. For the statistical purposes, the total immunoreaction scores of moderate and strong groups were classified as TET3^high^ group, while the negative and weak groups were classified as TET3^low^ group.

### Cell lines culture, transfection and Western blot assay

The human ESCC cell line (ECa109, KYSE510, KYSE150, TE-1) and human normal esophageal mucosa epithelium cell line (HEEC) were purchased from the Chinese Academy of Sciences. All the cell lines were cultured in DMEM, supplemented with 10% fetal bovine serum and 100 IU/mL penicillin/streptomycin in a humidified incubator, under 95% air and 5% CO_2_ at 37 °C.

The lentiviral vectors with TET3 was constructed by Hanyin Biotechnology Limited Company (Shanghai, China). The ov-TET3 lentiviral vectors was constructed according to NC_000002.12. One gRNA (AGTCTGATCGGACTCCGTAG) was designed for human TET3 against 0 to 1000 bp upstream of the first exon of TET3. We introduced dCas9-VP64 and pMS2-p65-HSF1 into cells by infection and established stable transductants. The sequence of normal control group lentiviral vectors was a non-targeted scrambled sequence. The constructed virus and Polybrene were added into the culture medium according to the instruction and co-cultured for 24 h. 48 h later, Puromycin was added to the culture medium to exclude the cells without successful transfection. Lipofectamine 2000 (Invitrogen, Carlsbad, USA) was applied for transient transfection. Predesigned siRNA duplexes were purchased from Genomeditech Company. The sequences of siRNA were listed in Additional file 1: Table S2. The normal control group was cells transfected with a non-targeted scrambled sequence. The transient transfection procedure was as follows: 4 × 10^4^ cells were seeded into a 24-well Corning culture plate at the previous day so that the adherent cell intensity could reach 60–80% at transfection. For each well, 2.5 μl siRNA + 50 μl Opti-mem and 1 μl lipo2000 + 50 μl Opti-mem were pre-mixed for 5 min at regular temperature. Next, siRNA and Lipo2000 were gently mixed, at a ratio of 1: 1, for 20 min at regular temperature. Subsequently, Pipetting 100 μl of the pre-mixed siRNA + lipo2000 into a well, and adding 400 μl culture medium free of penicillin/streptomycin to each well. After 6 h of transfection, culture medium was replaced with regular culture medium containing penicillin/streptomycin. Total RNA and total protein were extracted at 24 and 48 h after transfection, respectively.

The total protein was extracted with RIPA lysis buffer and protease inhibitor from the cell or tissues. For LPS or PBS pretreated group, cells were treated with LPS with corresponding concentration (1 or 8 μg/mL) or PBS for 48 h before total protein extraction. The Western blot assay was performed in the same way as previously described [21]. The related antibodies which were used to detect the expression of the related protein were listed in the Additional file 1: Table S1.

### RNA extraction and RT-qPCR analysis

The methods of total RNA extraction and reverse transcribe were performed in the same way with the previous work [21]. For LPS or PBS pretreated group, cells were treated with LPS with corresponding concentration (1 or 8 μg/mL) or PBS for 48 h before total RNA extraction. RT-qPCR was performed on an ABI 7500 thermocycler (Applied Biosystems, Foster City, CA). The fold change for each target gene relative to the control group was calculated using the ΔΔCt method. The primers for the genes detected with RT-qPCR were listed in the Additional file 1: Table S3.

### Fluorescence-activated cell sorter (FACS) analysis

FACS was performed on a CyAn ADP Analyzer (Beckman Coulter), and the data were analyzed with FlowJo software (Tree Star, Ashland, OR). The staining was performed following the manufacturer’s instructions. The related antibodies were listed in Additional file 1: Table S1 and applied according to the manufacturer’s instructions.

### ELISA assay

The ELISA assay was performed with the LPS-ELISA Kit (LanpaiBIO). The fresh ESCC tissues and para-cancerous tissues were treated, according to the protocol, to obtain the corresponding supernatants. The supernatants were subjected into the wells provided with the LPS-ELISA Kit, following the standard protocol as described in the Kit instruction.

### CCK-8, chemoresistance, colony-formation, transwell and sphere assay

For CCK-8 assay, cells proliferation and chemoresistance were detected according to the manufacturer’s instructions (CK04, Donjindo, Japan). The OD values were detected at 24 h, 48 h and 72 h after transfection, respectively. For the analysis of PTX function, at 24 h after transfection, the adherent cells were treated with PTX with 10 nM, 20 nM, 40 nM or 80 nM for another 24 h. And then the OD values were detected.

For colony-forming assay, 1.5 × 10^2^ cells were placed into a 60 mm cell culture dish. Cells of each group were cultured for 2 weeks. Then, cells were fixed with paraformaldehyde and stained with crystal violet. Colonies with more than 10 cells were counted from 5 random visual fields under microscope.

Transwell filters (Costar, Coring, NY) (6.5-mm insert, 8-mm polycar-bonate membrane) were chosen for Transwell assay. 5 × 10^3^ cells were harvested in 200 mL of serum-free medium and pipetted in the upper chambers of the plates. Then, 600 mL medium supplemented with 20% fetal bovine serum was added to the lower chambers of the plates. After incubation for 36 h, cells that had migrated from the upper chamber into the pores of the membrane insert were fixed with paraformaldehyde and stained with crystal violet. The cells were counted from 5 random visual fields under microscope.

For sphere assay, 2 × 10^3^ cells per well were cultured in ultralow attachment 24-well plates (Corning, USA) with the specific medium. The medium was DMEM/F12 supplemented with 10 ng/mL bFGF, 20 ng/mL EGF, B27 supplement (1×), 100 U/mL penicillin, and 100lg/mL streptomycin. The cells were cultured for 1 week to form primary spheres. Then, primary spheres were harvested and digested into single-cell suspensions, which were re-cultured in the specific medium for stem cells. After one-week culture, secondary spheres were recorded with microscopy and analyzed statistically. For LPS pretreated group, cells were treated with LPS for 1 week before the assay and kept on LPS stimulation till the end of assay.

### Tumor xenograft assay

4-week-old male nude mice were obtained from Slaccas Company. 2 × 10^6^ cells of each group were injected subcutaneously into either side of mice posterior flank. The normal Control group was implanted into the left posterior flank and the treatment group was implanted into the right of the same mouse. 3 weeks later, the mice were executed with cervical dislocation and the size of tumors were measured by caliper for statistical analysis. For LPS pretreated group, cells were treated with LPS for 1 week before the assay and the resuspension was also supplementary with LPS of corresponding concentration. The experiments complied with the ARRIVE guidelines and the care for the animals was in accordance with National Institutes of Health guide for the care and use of Laboratory animals.

### Genomic DNA isolation and dot blot assay

Cells genomic DNA was extracted with TIANamp Genomic DNA Kit (TIANGEN) and purified with Universal DNA purification Kit (TIANGEN). Then, the DNA was sonicated into fragments between 200 and 500 bp, and DNA concentration was measured with NanoDrop (Thermo Scientific). For Dot blot, nylon membrane was pre-wetted with methanol and following with TBST. Membrane was dried in regular temperature. DNA was diluted into specific concentration and spotted on membrane and placed under an ultraviolet lamp for 20 min to cross-link the DNA. Subsequently, the membrane was blocked with 5% milk in TBS-Tween for 1 h and then incubated with the specific antibody at 4 °C overnight. After incubation with the species appropriate HRP-conjugated secondary antibody for 1 h at regular temperature, the membrane was scanned by a Tanon 6100 scanner (Tanon, China). The 5hmC intensity was quantified by Image-J software (USA). For LPS or PBS pretreated group, cells were treated with LPS or PBS for 48 h before the assay.

### ChIP-qPCR and Nano-hmC-seal-seq

Chromatin immunoprecipitation quantitative real-time PCR (ChIP-qPCR) assay was performed according to a standard protocol. Briefly, cross-linked cell lysate was sonicated and subjected to immunoprecipitation reaction. The immunoprecipitated DNA was purified with Universal DNA purification Kit (TIANGEN). 1% of each sample volume was separated as the “Input”. HOXB2 antibody (1:400) was used as the primary antibody (Additional file 1: Table S1). The purified DNA was analyzed with qPCR. For LPS or PBS pretreated group, cells were treated with LPS or PBS for 48 h before the assay. The primer sequences are listed in Additional file 1: Table S3.

For Nano-hmC-Seal-seq, ov-control and ov-TET3 (lentiviral particles) cell lines with 3 independent samples were employed. Detailed information about extraction and quality inspection of gDNA samples, 5hmC-Seal library construction, sequencing and data processing was described elsewhere [22, 23]. Briefly, genomic DNA was extracted from cells using Quick-gDNA MicroPrep according to the manufacture’s instruction. After DNA purification, the captured DNA fragments were amplified by the Nextera kit. The sequencing steps were performed on the NextSeq 500 platform which contained a key pull-down step based on covalent chemistry. The raw 5hmC-Seal data was converted into clean reads through removing the reads with adapter, the reads whose proportion of “N” was over 10%, or the sequences less than 30 bp after quality trimming. The clean reads were aligned to the human genome reference [23]. The heatmap of signal distribution, K-means clustering, and genome-wide correlations were performed with deepTools software. The detection of 5hmC-enriched regions was performed with MACS. Peaks that were detected by all replicates were considered as high confident peaks. High quality alignments were summarized by counting overlaps with gene bodies. The raw data are available at the NCBI: PRJNA596911.

### Luciferase reporter assay

The cMYC and NANOG promoters (− 2000 to − 1 bp) were amplified by PCR and then cloned into the PHY-803 luciferase reporter vector through digestion, ligation of vector and target fragment, and sequencing (Hanyin, China). The primer sequences were listed in Additional file 1: Table S3. Cells were transfected with luciferase reporter genes, including internal control plasmid. 24 h after transfection, Firefly luciferase activities in cell lysates were measured with Luciferase Assay System (Promega), and normalized for transfection efficiency to the internal activity.

### Statistical analysis

SPSS was applied for statistical analysis. The data was noted as “mean ± standard deviation”. Chi-square test or Student t test was applied for two-sample comparisons. The overall survival (OS) curve was drawn with the Kaplan-Meier product limit estimator and analyzed with the log-rank test. The correlations between risk factors and protein expression level were analyzed with two-tailed Pearson test. All the tests at *p*-value < 0.05 were considered as significant difference.

## Results

### High concentration of LPS promotes the proliferation of ESCC

**Fig. 1:**
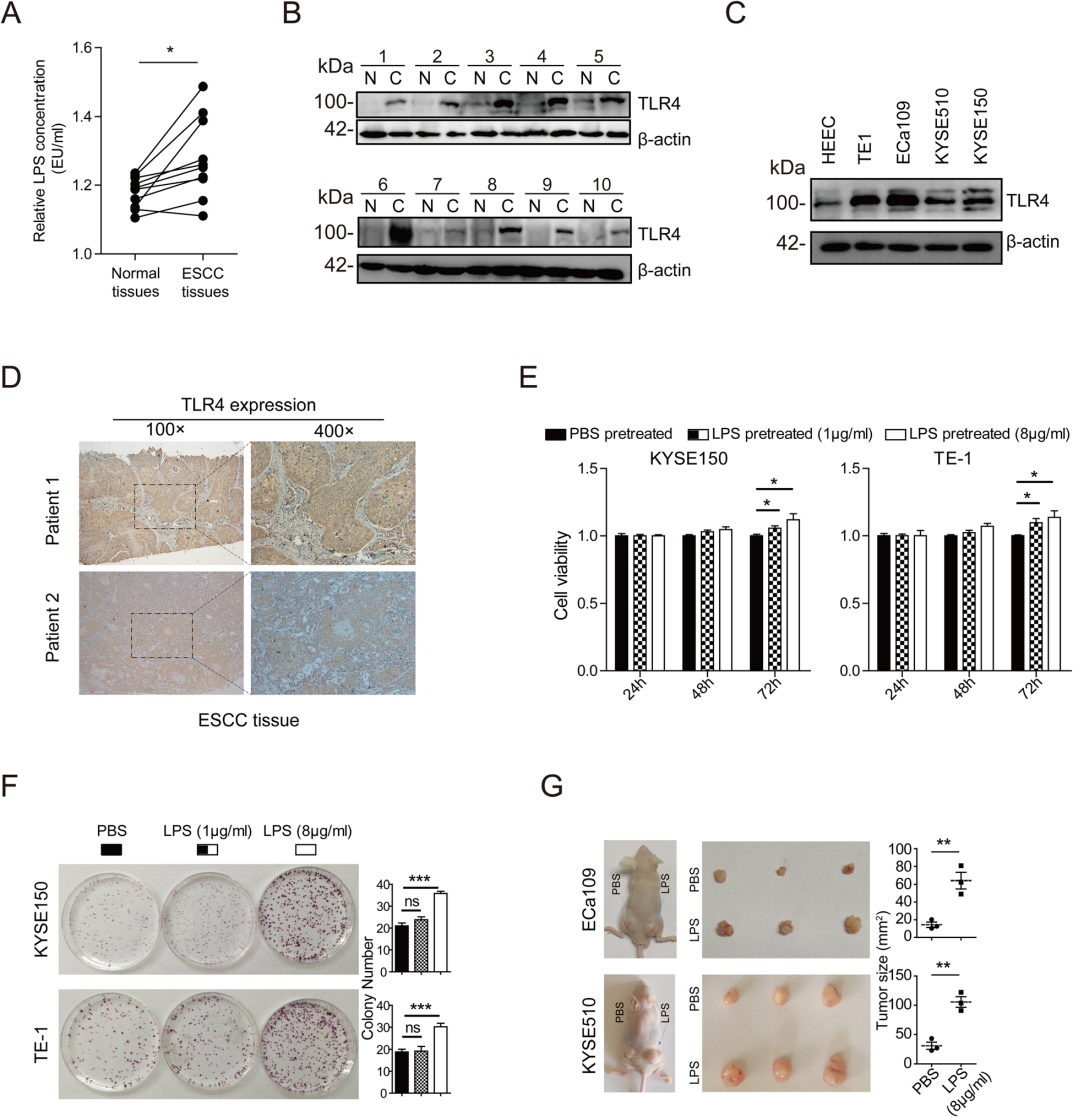
High concentration of LPS promoted the proliferation of ESCC. **a** ELISA was performed to detect the LPS concentration in normal esophageal epithelium tissues and ESCC. **b** Western blot was performed to detect the TLR4 expression in normal esophageal epithelium tissues and ESCC. **c** Western blot was performed to detect the TLR4 expression in normal esophageal epithelium cell line (HEEC) and ESCC cell lines (EC109, KYSE510, KYSE150 and TE-1). **d** IHC was applied to detect the location of TLR4 in ESCC. Typical images of two ESCC patients were presented. **e** CCK-8 was performed to assess the proliferation ability of ESCC cells with PBS or LPS stimulation. **f** colony-formation was performed to assess the proliferation ability of ESCC cells with PBS or LPS stimulation. **g** Tumor xenograft was applied to assess the proliferation ability of ESCC cells with PBS or LPS stimulation. PBS pretreatment group was implanted into the left posterior flank and LPS pretreatment group (8 μg/mL) was implanted into the right posterior flank of the same mouse. (ns: no significance, *p < 0.05, **p < 0.01, ***p < 0.001)

To investigate the potential significance of LPS stimulation in human ESCC progression, we firstly detected the LPS concentration in patients’ tissues. ELISA showed that, in 10 pairs of ESCC and para-cancerous tissues, LPS concentration was significantly up-regulated in ESCC tissues compared with the corresponding para-cancerous tissues (Fig. 1a). Next, Western blot demonstrated that the expression of TLR4, the receptor of LPS, was up-regulated in both ESCC tissues and cell lines, compared with the corresponding para-cancerous tissues or the human normal esophageal epithelial cell line HEEC (Fig. 1b and c). IHC results proved the cytoplasmic and membrane localization of TLR4 in ESCC tissue (Fig. 1d).

Further, we explored whether LPS made a difference in the progression of ESCC. CCK-8 results showed that LPS significantly accelerated the cells proliferation both in low (1 μg/mL) and high (8 μg/mL) dose (Fig. 1e). Colony-formation assay showed that low-dose LPS made no significant effect on the cells proliferation, while high-dose LPS significantly accelerated the cells proliferation (Fig. 1f). The tumor xenograft assay showed that high-dose LPS pretreatment could form significantly larger tumors in nude mice than PBS pretreatment (Fig. 1g).

### LPS stimulation induces the stemness of ESCC cells

**Fig. 2:**
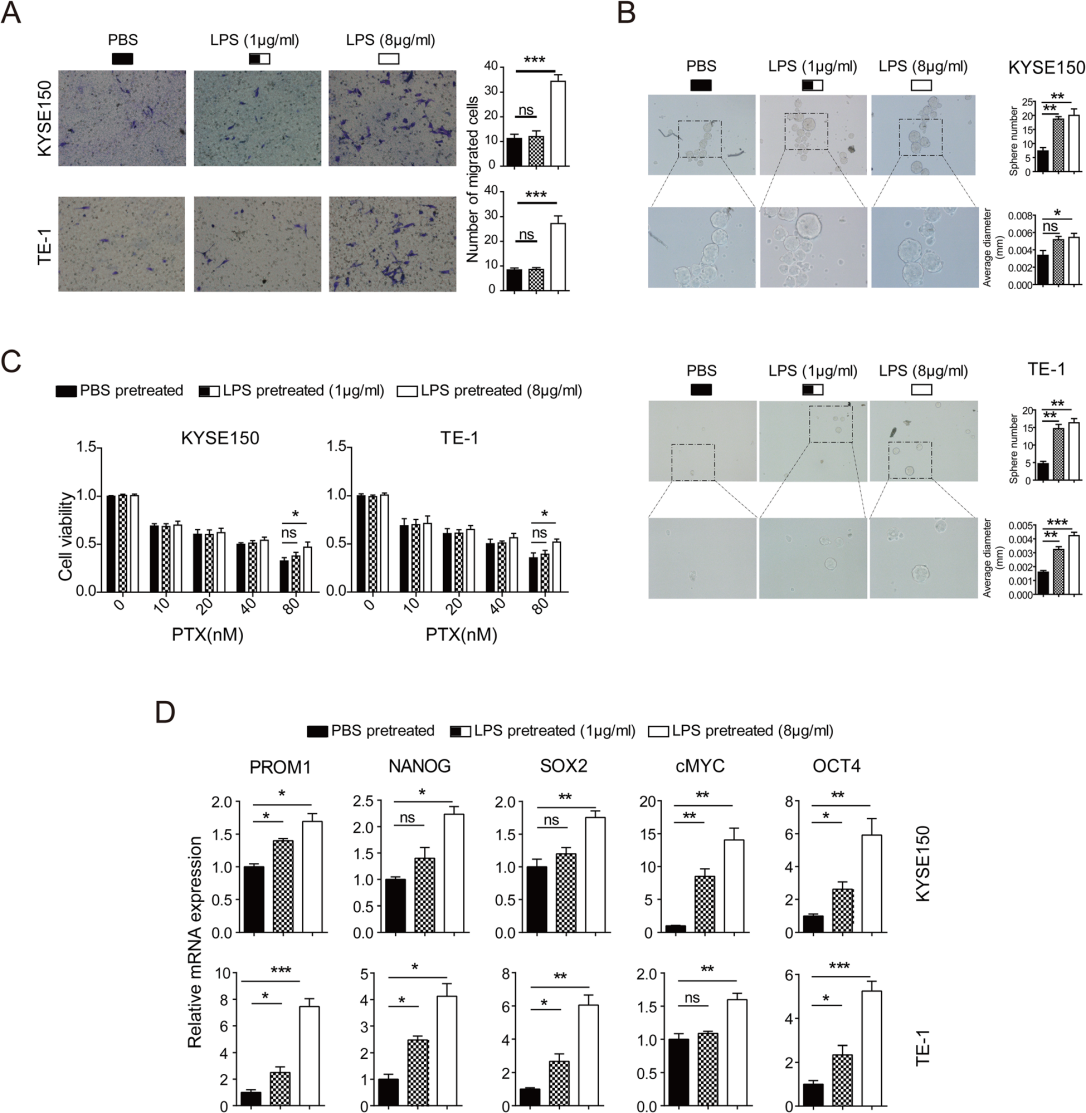
LPS stimulation induced the stemness of ESCC cells. **a** Transwell was performed to assess the migration ability of ESCC cells with PBS or LPS stimulation. **b** Sphere was performed to assess the sphere-formation ability of ESCC cells with PBS or LPS stimulation. **c** CCK-8 was performed to assess the chemoresistance ability of ESCC cells with PBS or LPS stimulation. **d** RT-qPCR was applied to detected stemness-related genes mRNA level in ESCC cells with PBS or LPS stimulation. (ns: no significance, *p < 0.05, **p < 0.01, ***p < 0.001)

To determine whether LPS exposure contributed to the progression of ESCC through the induction of ESCC cell stemness, we explored the function of LPS on ESCC cells’ stem cell characteristics, such as migration, sphere-formation, chemoresistance and stemness related gene expression. Transwell assay showed that low-dose LPS made no significant effect on the cells migration, while high-dose LPS significantly promoted the cells migration ability (Fig. 2a). Sphere assay showed that LPS pretreatment exhibited significantly greater effect on secondary sphere-formation capacity than PBS pretreatment did (Fig. 2b). Chemoresistance is another characteristic of CSCs [9]. Cells were treated with PTX with concentration gradient. CCK-8 showed that high-dose LPS significantly weakened the chemosensitivity to chemotherapeutic agents, indicating that LPS could induce chemoresistance of ESCC to PTX (Fig. 2c). Furthermore, stemness-related genes transcript levels, including PROM1 (also known named as CD133), NANOG, SOX2, cMYC and OCT4, were up-regulated with the simulation of LPS (Fig. 2d). These data demonstrates that LPS stimulation induces the stemness of ESCC cells.

### LPS induces TET3 over-expression in ESCC

**Fig. 3:**
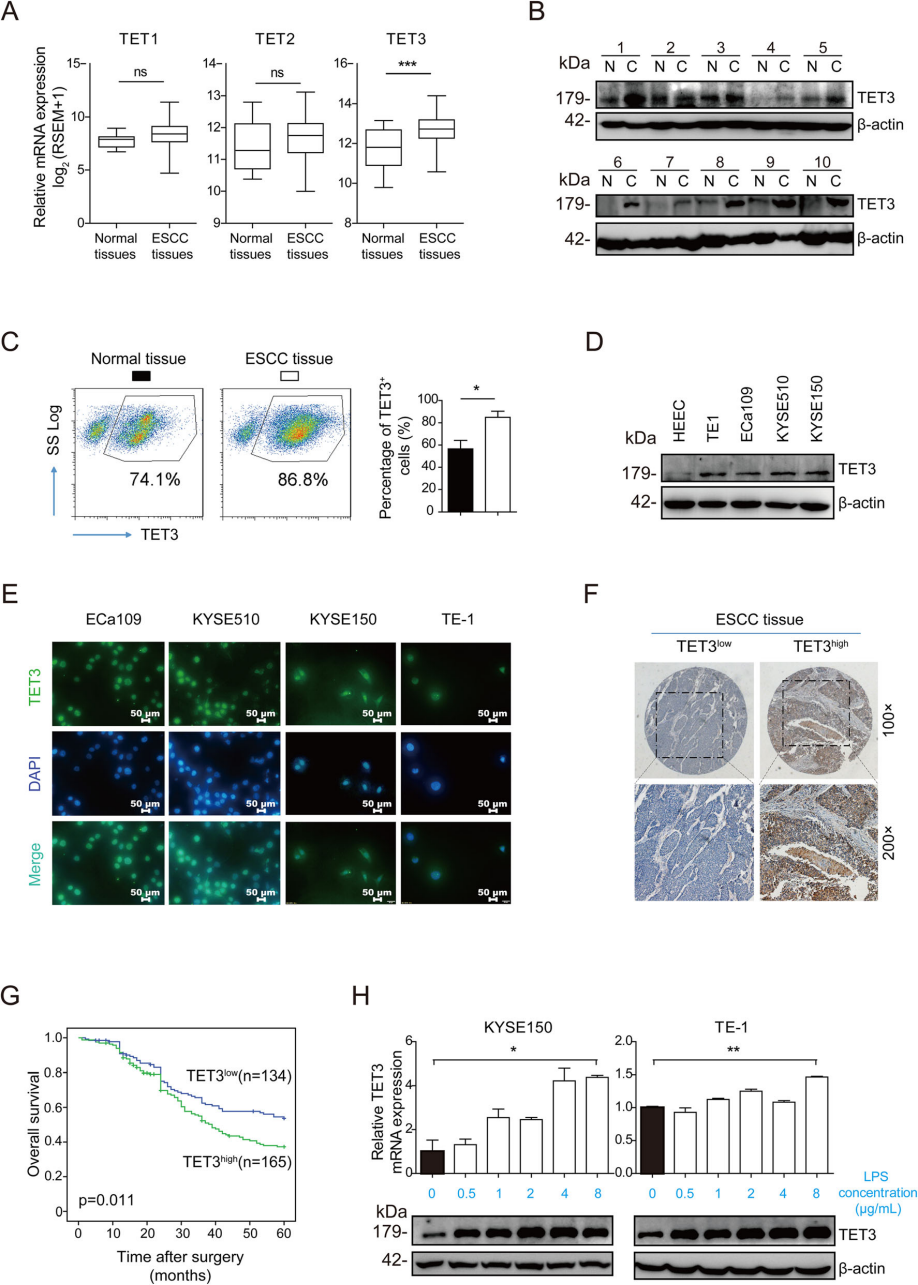
LPS induced TET3 over-expression in ESCC. **a** The TET protein family mRNA expression in ESCC was analyzed based on TCGA database. **b** Western blot was performed to detect the TET3 expression in normal esophageal epithelium tissues and ESCC. **c** FACS was performed to detect the TET3 expression in patients’ normal esophageal epithelium tissues and ESCC tissues. The plots of a representative pair of cancerous and para-cancerous tissues were shown in the left column, and the statistical result of total patients’ data was shown in the right column. **d** Western blot was performed to detect the TET3 expression in normal esophageal epithelium cell line (HEEC) and ESCC cell lines (EC109, KYSE510, KYSE150 and TE-1). **e** IF was performed to detect the location of TET3 in ESCC cell lines. **f** IHC was applied to detect TET3 expression in ESCC tissues from 299 enrolled patients. The typical images of TET3^low^ and TET3^high^ were exhibited. **g** Kaplan-Meier product limit estimator was applied to draw overall survival (OS) curves of 299 enrolled ESCC patients, compared according to TET3 expression level. **h** RT-qPCR and Western blot were performed to detect the TET3 expression in ESCC cell lines with the stimulation of LPS. (ns: no significance, *p < 0.05, **p < 0.01, ***p < 0.001)

It is well known that DNA methylation plays an important role in the regulation of tumor cells’ stemness. Various methylases have been reported to regulate the stemness of various tumors [13]. Considering DNA methylation levels are maintained by the dynamic balance between methylases and demethylases [11], we focused on the role of demethylases, TET family, in the regulation of ESCC cell stemness. We analyzed the TET family members, TET1/2/3, expression in ESCC according to the TCGA database and found that only TET3 was significantly over-expressed, while no significant difference existed in TET1 or TET2 expression compared with the para-cancerous tissues (Fig. 3a, Additional file 2: Table S4). We speculated that TET3 might contribute to the induction of ESCC cell stemness and the progression of ESCC. RT-qPCR, Western blot and FACS data proved that TET3 expression, both in RNA and protein level, were over-expressed in ESCC tissues and cell lines, compared with para-cancerous tissues or normal esophageal mucosa epithelium cell line (Fig. 3b-d, Additional file 1: Figure S1). Immunofluorescence and IHC data showed that TET3 located in cytoplasm and nucleus (Fig. 3e). In the analysis of survival, we applied IHC in tissue microarray and uncovered that patients with high level of TET3 suffered shorter 5-year OS than the patients with low level of TET3 did (Fig. 3f and g). The analysis of correlationship between TET3 and clinicopathological features showed that male patients and poor tumor differentiation were significantly associated with TET3 high expression level (Table 1). In univariate analysis, tumor size, TNM stage and TET3 level were correlated with 5-year OS (Table 2). The survival analysis showed that, when patients were distributed into high/low differentiation groups or high/low stage groups, high level of TET3 led to a shorter 5-year OS than low level of TET3 did in each distribution (Additional file 1: Figures S2A–S2D). In multivariate analysis, TNM stage and TET3 expression served as the independent prognosis factors for ESCC patients (Table 2).

**Table 1:** Correlation between TET3 and clinicopathological features in 299 ESCC patients

Variables	NO. of patients	TET3 expression		*P*
		Low	High	
Age				
< 60	156	72(46)	84(54)	0.627
≥ 60	143	62(43)	81(57)	
Gender				
Male	231	95(41)	136(59)	0.018
Female	68	39(57)	29(43)	
Smoking status				
Smokers	163	68(42)	95(58)	0.238
Non-smokers	136	66(49)	70(51)	
Differentiation				
I	19	13(68)	6(32)	0.033
II-III	280	121(43)	159(57)	
Tumor invasion depth				
T1–2	117	58(50)	59(50)	0.185
T3–4	182	76(42)	106(58)	
Tumor stage				
I-II	189	85(45)	104(55)	0.943
III-IV	110	49(45)	61(55)	
Lymph node metastasis				
Yes	178	86(48)	92(52)	0.140
No	121	48(40)	73(60)	
Tumor size				
< 3 cm	110	57(52)	53(48)	0.063
≥ 3 cm	189	77(41)	112(59)

**Table 2:** Univariate and Multivariate analysis of factors associated with OS

Variables	Univariate Analysis			Multivariate Analysis		
	HR	95% CI	*P*	HR	95% CI	*P*
Age (≥60 vs. < 60)	1.224	0.893–1.678	0.208			
Gender (female vs. male)	0.747	0.504–1.108	0.147			
Smoking status (smokers vs. non-smokers)	1.192	0.867–1.639	0.280			
Tumor size(≥3 cm vs. < 3 cm)	1.859	1.307–2.645	0.001	1.426	0.988–2.057	0.058
Tumor stage (III-IV vs. I-II)	2.420	1.760–3.327	< 0.001	2.233	1.606–3.105	< 0.001
TET3 level (high vs. low)	1.510	1.091–2.090	0.013	1.479	1.066–2.051	0.019

**Abbreviations:***OS* overall survival, *95%CI* 95% confidence interval, *HR* hazard ratio

To investigate whether LPS could regulate TET3 expression, we performed RT-qPCR and Western blot, demonstrating that LPS stimulation could up-regulate TET3 expression, RNA and protein level, in a concentration gradient manner. Thus, we speculated LPS might induce the stemnss of ESCC possibly through the up-regulation of TET3 (Fig. 3h).

### TET3 contributes to inducing the stemness of ESCC cells

**Fig. 4:**
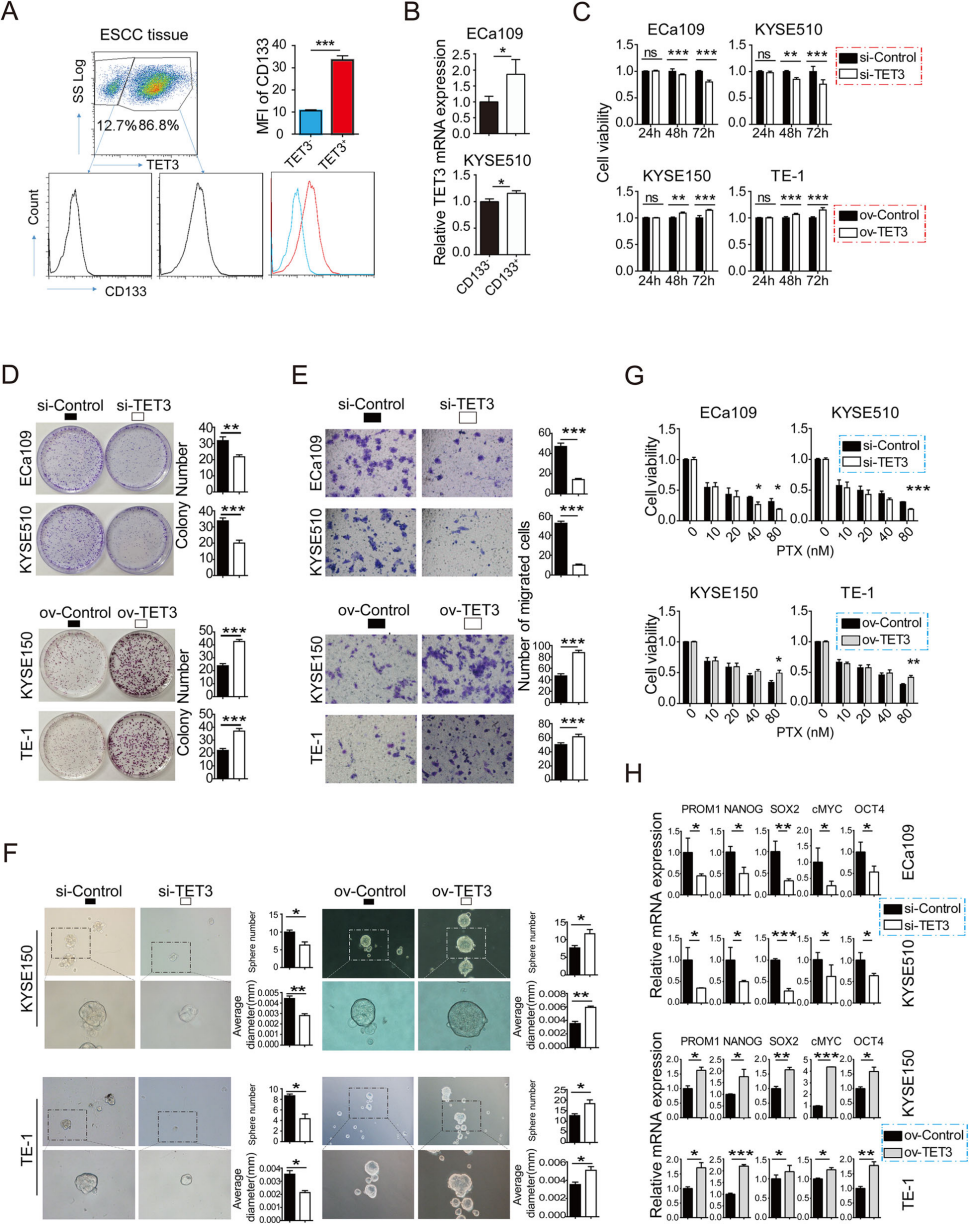
TET3 contributed to inducing the stemness of ESCC cells. **a** FACS was performed to detect the CD133 expression in TET3-negative and TET3-positive group in ESCC patients’ tissues. The plots of a representative ESCC tissue was shown, and the statistical result of a total patients’ data was shown in the upper right corner. **b** RT-qPCR was performed to TET3 mRNA level in CD133-positive and CD133-positive group in ESCC tissues. **c** CCK-8 was applied to assess the proliferation ability of ESCC cells with knockdown or overexpression of TET3. **d** Colony-formation was applied to assess the proliferation ability of ESCC cells with knockdown or overexpression of TET3. **e** Transwell was employed to assess the migration ability of ESCC cells with knockdown or overexpression of TET3. **f** Sphere was applied to assess the sphere-formation ability of ESCC cells with knockdown or overexpression of TET3. **g** CCK-8 was performed to assess the chemoresistance ability of ESCC cells with knockdown or overexpression of TET3. **h** RT-qPCR was applied to detected stemness-related genes mRNA level in ESCC cells with knockdown or overexpression of TET3. (ns: no significance, *p < 0.05, **p < 0.01, ***p < 0.001)

Given TET3 could be up-regulated with the stimulation of LPS, which also induced the stemness of ESCC cells, we sought to investigate whether TET3 could contribute to inducing the stemness of ESCC cells. FACS data showed that in ESCC tissues, CD133, an acknowledged and classical stem cell marker [24], expression was significantly higher in TET3-high cells than in TET3-low cells (Fig. 4a). We further sorted CD133-positive and CD133-negative cells in ESCC cell lines with FACS. RT-qPCR showed that TET3 expression was significantly higher in CD133-positive cells than in CD133-negative cells (Fig. 4b). These data indicates that TET3 expression level is positively correlated with CD133 expression level.

Next, we got down to investigate the role of TET3 in the stem cell characteristics. The knockdown (si-control vs si-TET3) and overexpression (ov-control vs ov-TET3) of TET3 in cell lines were verified with RT-qPCR and Western blot (Additional file 1: Figures S3A and S3B). CCK-8, Colony-formation and Sphere showed that knockdown of TET3 inhibited cells proliferation, migration, sphere-formation and chemoresistance ability, while overexpression of TET3 promoted cell proliferation, migration, sphere-formation and chemoresistance ability (Fig. 4c-g). Meanwhile, cells with overexpression of TET3 could form significantly larger tumors in nude mice than the normal control group did in vivo (Additional file 1: Figure S4). In addition, knockdown of TET3 downregulated stemness-related genes transcript levels, while overexpression of TET3 upregulated stemness-related genes transcript levels including (Fig. 4h). These data indicates that TET3 contributes to inducing the stemness of ESCC cells, which might be triggered by LPS stimulation.

### HOXB2 serves as the downstream target of TET3 and participates in inducing the stemness of ESCC cells

**Fig. 5:**
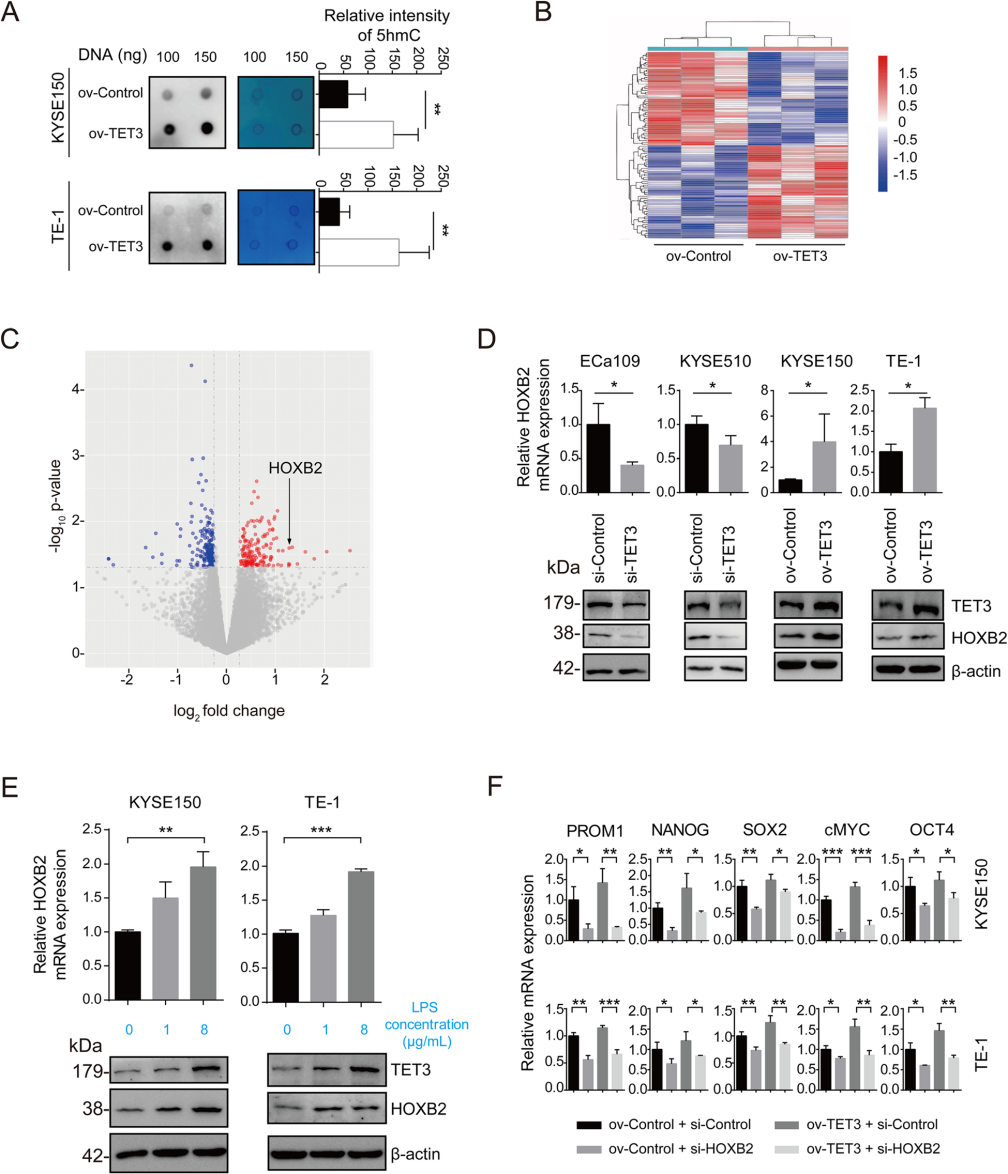
HOXB2 served as the downstream target of TET3 and participated in inducing the stemness of ESCC cells. **a** Dot-blot was performed to detect the 5hmC DNA level of ESCC cells with overexpression of TET3 (left panel). The right panel shows the membrane stained with methylene blue as an internal reference. **b** Heat map of the most up-regulated (red) and down-regulated (blue) genes (based on *p* value) in TET3-overexpression group compared with Control group analyzed with Nano-hmC-Seal-seq. **c** Scatterplot of *p* values for all genes in both groups analyzed with Nano-hmC-Seal-seq. Significantly up-regulated and down-regulated proteins in TET3-overexpression cells were highlighted in red and blue, respectively. **d** RT-qPCR and Western blot were performed to detected HOXB2 expression in ESCC cells with knockdown or overexpression of TET3. **e** RT-qPCR and Western blot were performed to detected HOXB2 expression in ESCC cells with PBS or LPS stimulation. **f** RT-qPCR was performed to detect stemness-related genes mRNA level in ESCC cells with knockdown of HOXB2 or/and overexpression of TET3. (ns: no significance, *p < 0.05, **p < 0.01, ***p < 0.001)

We explored the downstream target of TET3 to uncover the mechanism how TET3 induced the stemness of ESCC cells. For the reason that TET3 served as a demethylase which catalyzed the oxidation of 5mC to 5hmC in DNA [15], we wondered the difference in 5hmc levels of downstream genes when TET3 was overexpressed. Dot-blot data confirmed that global 5hmC levels were significantly increased with the overexpression of TET3 and stimulation of LPS in ESCC cell lines (Fig. 5a and Additional file 1: Figure S5). Next, we performed the Nano-hmC-Seal-seq to analyze the 5hmC level difference between ov-control and ov-TET3 cells. According to Nano-hmC-Seal-seq data, we identified 158 genes with increase in 5hmC levels (≥1.2 fold) and another 183 genes with decrease in 5hmC levels (≥1.2 fold), resulted from TET3 overexpression (Fig. 5b and Additional file 3: Table S5). Considering transcriptional regulation is one of the main process for the induction and maintenance of the stemnss of cancer cells, we paid attention to the transcription factors in the differential genes and identified HOXB2, a member of a classical transcription factor: Homeobox, might be the potential downstream target of TET3 (Fig. 5c). RT-qPCR and Western blot showed that HOXB2 expression was positively correlated with TET3 expression (Fig. 5d), and also could be upregulated with LPS stimulation (Fig. 5e). Meanwhile, knockdown of TET3 could reverse the up-regulation of HOXB2 induced with LPS (Additional file 1: Figure S6A). Furthermore, knockdown of HOXB2 had no influence in TET3 expression (Additional file 1: Fig. S6B). In addition, RT-qPCR showed that knockdown of HOXB2 deceased stemness-related genes expression in ov-control groups and ov-TET3 groups (Fig. 5f). Thus, we confirms that HOXB2 serves as the downstream target of TET3.

### LPS activates p38/ERK-MAPK pathway to promote stemness-related gene transcription

**Fig. 6:**
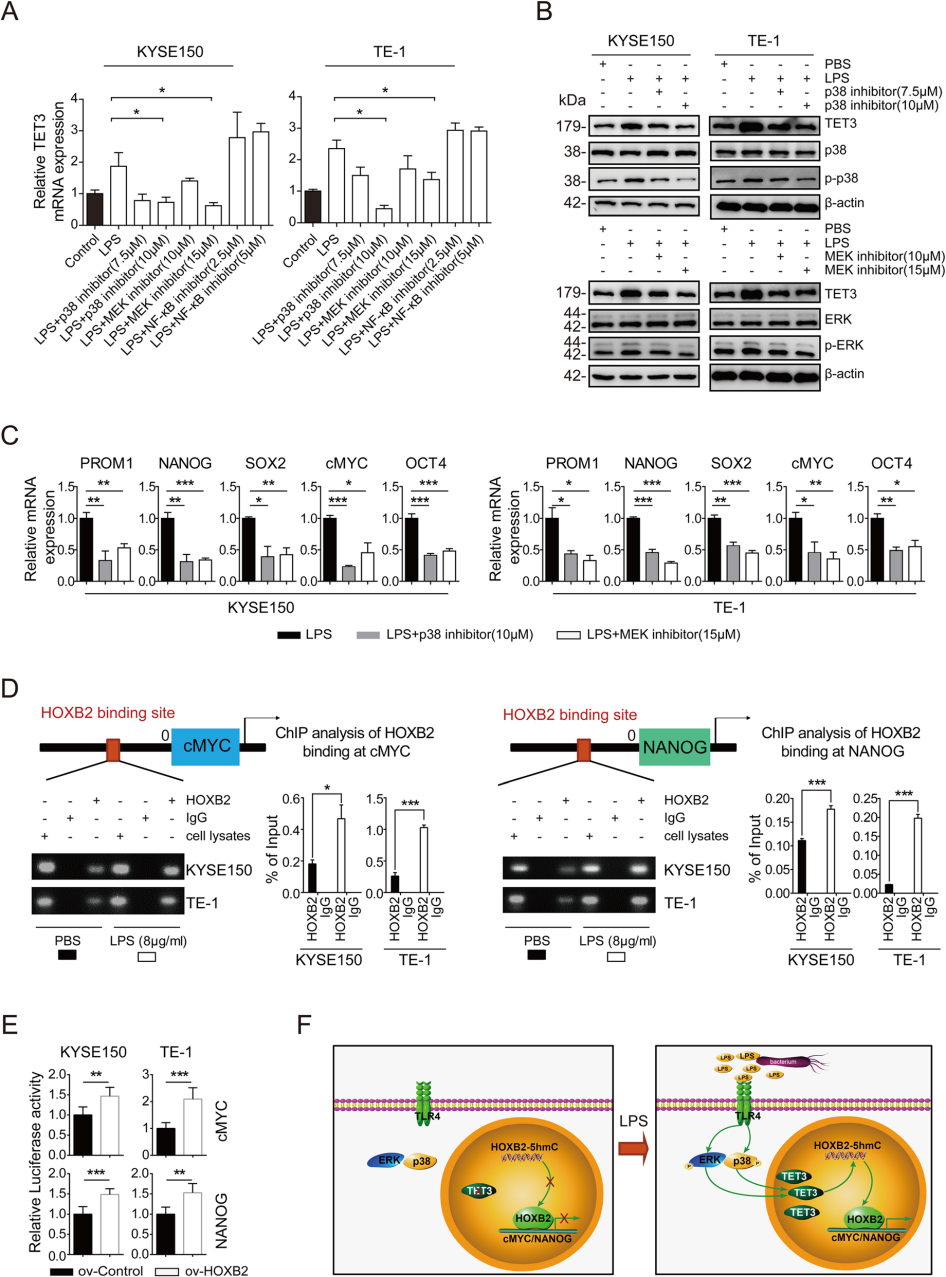
LPS activated p38/ERK-MAPK pathway to promote stemness-related gene transcription. **a** RT-qPCR was performed to detect TET3 mRNA level. ESCC cells were pretreated with p38, MEK and NF-κB inhibitors for 30 min, whereas the control groups were treated with DMSO instead. Cells were then stimulated with LPS (1 μg/mL) for 24 h, whereas the control groups were stimulated with PBS instead. **b** Western was performed to detect TET3 protein level. ESCC cells were pretreated with p38 and MEK inhibitors for 30 min, whereas the control groups were treated with DMSO instead. Cells were then stimulated with LPS (1 μg/mL) for 48 h, whereas the control groups were stimulated with PBS instead. **c** RT-qPCR was performed to detect stemness-related genes mRNA level in ESCC cells with LPS stimulation, together with p38 or MEK inhibitor. **d** ChIP-qPCR was performed to detect the binding of HOXB2 to cMYC and NANOG in ESCC cells with PBS or LPS stimulation. **e** Luciferase was performed to study the cMYC and NANOG promoter activity regulated by HOXB2. **f** A model graph summarized the mechanism for epigenetic induction of the stemness of ESCC cell through a LPS-TET3-HOXB2 signaling axis. LPS activated p38/ERK-MAPK pathway to up-regulate TET3 expression. Then TET3 increased 5hmC level in HOXB2 gene region, which promoted stemness-related gene transcription, thereby inducing the stemness of ESCC cells (ns: n (ns: no significance, *p < 0.05, **p < 0.01, ***p < 0.001)

To investigate the mechanism how LPS induced the stemness of ESCC cells through the activation of LPS-TET3-HOXB2 signaling axis, we first explored how LPS upregulated TET3 expression. It has been reported that MAPK and NF-κB signaling pathways were two most classical pathways triggered with LPS [25]. We employed p38 inhibitor SB202190, MEK inhibitor U0126 and NF-κB inhibitor BAY11–7082 to pretreat the cells before the stimulation of LPS. Then RT-qPCR was applied to detect TET3 expression and showed that SB202190 and U0126 decreased TET3 expression significantly, while BAY11–7082 failed to inhibit the LPS stimulation on TET3 expression (Fig. 6a). Western blot confirmed the consistent results (Fig. 6b). These data indicated that p38/ERK-MAPK signaling pathway might participate in the function of LPS stimulation on TET3 expression. We further proved that SB202190 and U0126 successfully blocked the LPS simulation of stemness-related genes expression (Fig. 6c). Therefore, we drew the conclusion that LPS activated p38/ERK-MAPK signaling pathway to upregulate TET3 expression and induce the stemness of ESCC cells.

As HOXB2 served as a transcription factor [26], we further hypothesized that HOXB2 may be involved in regulating the transcription of stemness-related genes. Through the sequence analysis of the stemness-related genes promoters with the Jaspar database, which is a tool for the prediction of the binding sites for transcription factors and promoters, we discovered potential HOXB2 binding sites in cMYC and NANOG promoters. ChIP-PCR data proved the recruitment of HOXB2 to the cMYC and NANOG promoters regions in ESCC cells lines. LPS pretreatment group (8 μg/mL), in which cells were overexpressed with TET3 and HOXB2 expression, showed higher level of HOXB2 binding to both cMYC and NANOG promoters regions than PBS pretreatment group did (Fig. 6d). Luciferase data demonstrated that the transcriptional activity of the cMYC and NANOG were greatly enhanced by overexpression of HOXB2 (Fig. 6e). These data indicates that LPS activates p38/ERK-MAPK pathway to promote stemness-related gene transcription through LPS-TET3-HOXB2 signaling axis, thereby inducing stemness and promoting ESCC progression (Fig. 6f).

## Discussion

Chronic inflammation has been recognized as one of the most important factors in promoting tumorigenesis and development [27, 28]. LPS, as a metabolite of Gram-negative bacteria, is a common proinflammatory factor [7]. A large number of studies have proved that LPS participated in stimulating the progression of several types of tumors, He and colleagues found that LPS promotes the migration of oral squamous cell carcinoma by activating the epithelial-mesenchymal transition signaling pathway [29]. Jain and colleagues confirmed that LPS promoted the distant metastasis of prostate cancer by activating NF-κB signaling pathway, and further accelerated the metastasis under the stimulation of dexamethasone [30]. In addition to the effects of LPS on migration and metastasis, some researchers have reported the effect of LPS on tumor proliferation. Li and colleagues verified that LPS activated TLR4/MD-2 signaling pathway through the induction of CXCR7 expression to promote gastric cancer proliferation and migration [31].

LPS also has a regulatory effect on the induction and maintenance of stemness of tumor cells. Lai and colleagues confirmed that LPS could participate in the maintenance of stemness of liver cancer cells by activating NF-κB/HIF-1α signaling pathway [32]. Similar results were also found by Li et al., who confirmed that LPS promoted the proliferation of mouse liver stem cells and suppressed their regular differentiation. Long-term exposure of liver stem cells to LPS increased the risk of tumor formation [33]. The regulation way of LPS on these tumor cells and the induction of stemness of cells were consistent with the results of this study.

TET protein family, serving as demethylases, was first discovered to play a role in cancer in 2003. TET1 was proved to be fused to the mixed lineage of leukemia gene in a case of pediatric AML containing the t(10;11) (q22;q23) [34]. Later, TET1 was verified to promote cell proliferation and induce leukemia by activating the Hoxa9/Meis1/Pbx3 signaling pathway [35]. However, TET2 was reported to play a regulatory role in myeloproliferative neoplasms and myelodysplastic syndromes in the form of functional deletion mutations [36], whereas TET1 and TET3 were rarely characterized of functional deletion mutations to function in hematological tumors [37].

With the deepening of research, the regulation of the TET protein family in solid tumors was gradually revealed. Early reports consistently believed that TET family members acted as tumor suppressor genes. Ye et al. demonstrated that TET3 demethylated the miR-30d precursor gene promoter to block TGF-β1 inducing epithelial-mesenchymal transition, thereby inhibiting ovarian cancer progression [38]. Zhu and colleagues confirmed that TET2 inhibited the carcinogensis and progression of breast tumors by regulating caspase-4 [39]. However, recent studies also revealed the tumor-promoting role of TET protein family in cancers. Pan and colleagues demonstrated that Tet2 promoted the progression of mouse melanoma by maintaining the myeloid cells that exerted immunosuppressive functions in the tumor microenvironment [40]. Deng et al. proved that TET1, TET2 and TET3 transcription levels were upregulated in gastric cancer tissues, and the high expression of TET1, TET2 and TET3 were significantly associated with the poor overall survival of patients. This study also revealed that TET family serving as a ceRNA significantly to enhance EZH2 expression by competitively binding to miR-26, thereby exerting carcinogenic effects on gastric cancer [41].

In this study, according to the TCGA database, TET3 expression in ESCC tissues was significantly higher than that in para-cancerous tissues, neither TET1 nor TET2 expression showed significant difference between ESCC tissues and para-cancerous tissues. Hence, we speculated that TET3 played a dominant role, compared with TET1 and TET2, in the progression of ESCC. And we also confirmed that TET3 was upregulated in ESCC tissues and cell lines with experiments. Considering the 5hmC level was regulated with the entire TET family and even other factors in the tumorimmune microenvironment, we believed that the exact mechanism of TET family and 5hmC functioning on tumors, in global level or some particular loci level, need further comprehensive sequencing and investigation.

In addition, the TET protein family has also been found to be involved in the regulation of CSCs. Prasad and colleagues demonstrated that hypoxic environment could induce the demethylation in glioma cells by overexpression of TET1 and TET3, then promoted the stemness of tumor cells by upregulating OCT4 and NANOG expression [42]. However, in ESCC, no study has reported the effect of the TET family on the stemness of tumor cells yet. Our data demonstrated that TET3 could promote the proliferation, migration, chemoresistance, Sphere-formation, and positively regulate the expression level of stemness-related genes, which acted together to induce the cel stemness and promote the progression of ESCC.

LPS classically triggers downstream NF-κB and MAPK pathway in the innate immune system [25]. In previous studies, Xie and colleagues verified that LPS promoted the proliferation and invasive ability of colorectal cancer cells by activating TLR4/p65-NF-κB in an inflammatory environment [43]. Li and colleagues confirmed that LPS, produced from the gastric infection environment, promoted the progression of gastric cancer, with the underlying mechanism of the activation of LPS-NF-κB-PD-L1 signal axis [44]. Our results showed that LPS upregulated TET3 expression mainly through the co-activation of p38/ERK- MAPK signaling pathways. Furthermore, activation of the MAPK signaling pathway is also one of the important pathways for the induction of the stemness of tumor cells. Xu and colleagues found that alcohol could enhance the tumor-promoting role of breast cancer stem-like cells through the activation of ErbB2/p38γ-MAPK signaling pathway [45]. Xie and colleagues confirmed that long-term stimulation of tobacco in liver cells was prone to induce the stemness properties of liver cancer cells by activating the IL-33/p38 signal axis [46]. Berardi and colleagues confirmed that laminin regulated the function of stem cell populations in mouse breast cancer cells via the MAPK/ERK signaling pathway [47]. We tried to explore the downstream target of TET3 and applied the Nano-hmC-Seal-seq to figure out HOXB2 as the potential target. As a transcription factor, HOXB2 binds to the promoter of its target genes and regulates their expression, which subsequently results in a cascade of biological events [26]. Our data proved that HOXB2 could directly bind to the promoter region of cMYC and NANOG, thereby activating the transcription of cMYC and NANOG. Zhan and colleagues also achieved similar results that TR3 promoted the stem-like properties of gastric cancer cells through the transactivation of NANOG [48]. Thus, we drew the conclusion that LPS induced the stemness of ESCC cells through a LPS-TET3-HOXB2 signaling axis.

## Conclusions

To summarize, this study uncovered evidence for a new functional role of LPS-TET3-HOXB2 signaling axis in promoting the stemness of ESCC cells. Exploring effective methods to block LPS-TET3-HOXB2 signaling axis might be a potential therapeutic strategy to prevent or postpone the progression of ESCC.

## Supplementary information

**Additional file 1:Figure S1.** (A) Flow cytometry gating strategies and representative plots. (B) RT-qPCR was performed to detected TET3 mRNA level in ESCC tissues and para-cancerous tissues. (C) RT-qPCR was performed to detected TET3 mRNA level in ESCC cell lines and normal esophageal mucosa epithelium cell line. **Figure S2.** Kaplan-Meier product limit estimator was applied to draw overall survival (OS) curves of 299 enrolled ESCC patients, compared according to TET3 expression level, in stage I-II (A), stage III-IV (B), grade I (C) and grade II-III (D). **Figure S3.** The knockdown (si-control vs si-TET3) and overexpression (ov-control vs ov-TET3) of TET3 in cell lines were verified with RT-qPCR (A) and Western blot (B). **Figure S4.** Tumor xenograft was applied to assess the proliferation ability of ESCC cells influenced by TET3 expression. ov-Control group was implanted into the left posterior flank and ov-TET3 group was implanted into the right posterior flank of the same mouse. (ns: no significance, *p < 0.05, **p < 0.01, ***p < 0.001). **Figure S5.** Dot-blot was performed to detect the 5hmC DNA level of ESCC cells with with PBS or LPS stimulation (left panel). The right panel shows the membrane stained with methylene blue as an internal reference. **Figure S6.** (A) RT-qPCR and Western blot were applied to detect HOXB2 expression upon LPS stimulation and knockdown of TET2. (B) RT-qPCR and Western blot were applied to detect whether knockdown of HOXB2 influenced TET3 expression. (ns: no significance, *p < 0.05, **p < 0.01, ***p < 0.001). **Table S1.** Antibodies, inhibitors and other reagents. **Table S2.** Sequences for siRNA (5′-3′). **Table S3.** Primer sequences for PCR (5′-3′).

**Additional file 2: Table S4.** The TCGA data for genes expression in ESCC patients.

**Additional file 3: Table S5.** The differential genes detected with Nano-hmC-Seal-seq.

## Data Availability

All data generated or analyzed during this study are included in this article.

## Abbreviations

CI: Confidence interval
ESCC: Esophageal squamous cell carcinoma
HOXB2: Homeobox B2
HR: Hazard ratio
OS: Overall survival
PTX: Paclitaxel
TET: Ten-eleven-translocation
TMN: Tumor-node-metastasis
