# Supplementary material for: Epigenetic induction of tumor stemness via the lipopolysaccharide-TET3-HOXB2 signaling axis in esophageal squamous cell carcinoma
Source: Cell Commun Signal. 2020 Feb 3;18:17. doi: 10.1186/s12964-020-0510-8 (PMC6998358; doi:10.1186/s12964-020-0510-8)
Supplement: Supplementary file 2 — Additional file 1:Figure S1. (A) Flow cytometry gating strategies and representative plots. (B) RT-qPCR was performed to detected TET3 mRNA level in ESCC tissues and para-cancerous tissues. (C) RT-qPCR was performed to detected TET3 mRNA level in ESCC cell lines and normal esophageal mucosa epithelium cell line. Figure S2. Kaplan-Meier product limit estimator was applied to draw overall survival (OS) curves of 299 enrolled ESCC patients, compared according to TET3 expression level, in stage I-II (A), stage III-IV (B), grade I (C) and grade II-III (D). Figure S3. The knockdown (si-control vs si-TET3) and overexpression (ov-control vs ov-TET3) of TET3 in cell lines were verified with RT-qPCR (A) and Western blot (B). Figure S4. Tumor xenograft was applied to assess the proliferation ability of ESCC cells influenced by TET3 expression. ov-Control group was implanted into the left posterior flank and ov-TET3 group was implanted into the right posterior flank of the same mouse. (ns: no significance, *p < 0.05, **p < 0.01, ***p < 0.001). Figure S5. Dot-blot was performed to detect the 5hmC DNA level of ESCC cells with with PBS or LPS stimulation (left panel). The right panel shows the membrane stained with methylene blue as an internal reference. Figure S6. (A) RT-qPCR and Western blot were applied to detect HOXB2 expression upon LPS stimulation and knockdown of TET2. (B) RT-qPCR and Western blot were applied to detect whether knockdown of HOXB2 influenced TET3 expression. (ns: no significance, *p < 0.05, **p < 0.01, ***p < 0.001). Table S1. Antibodies, inhibitors and other reagents. Table S2. Sequences for siRNA (5′-3′). Table S3. Primer sequences for PCR (5′-3′). [file 12964_2020_510_MOESM2_ESM.docx]

Supplementary Figure S1


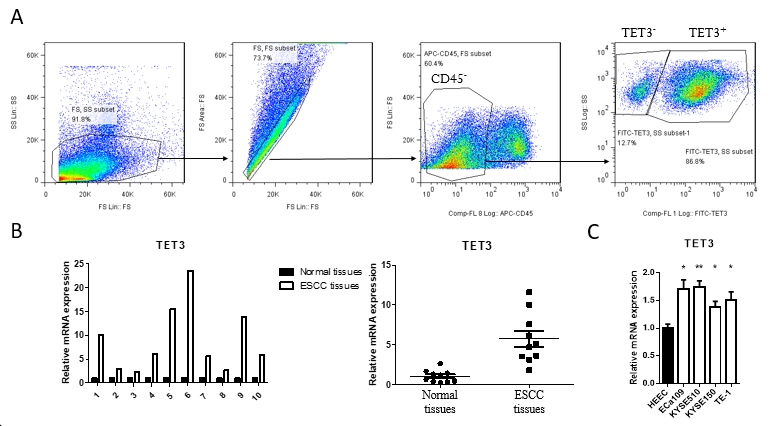


**Supplementary Figure S1** (A) Flow cytometry gating strategies and representative plots. (B) RT-qPCR was performed to detected TET3 mRNA level in ESCC tissues and para-cancerous tissues. (C) RT-qPCR was performed to detected TET3 mRNA level in ESCC cell lines and normal esophageal mucosa epithelium cell line.

Supplementary Figure S2


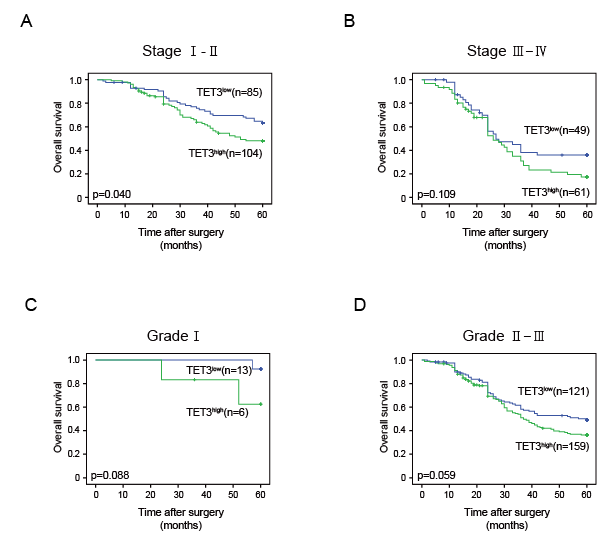


**Supplementary Figure S2** Kaplan-Meier product limit estimator was applied to draw overall survival (OS) curves of 299 enrolled ESCC patients, compared according to TET3 expression level, in stage Ⅰ-Ⅱ (A), stage Ⅲ-Ⅳ (B), grade Ⅰ (C) and grade Ⅱ-Ⅲ (D).

Supplementary Figure S3


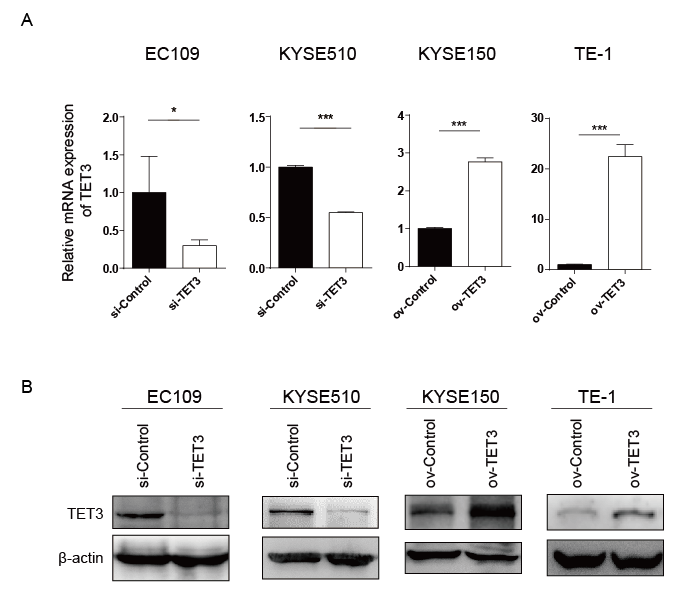


**Supplementary Figure S3** The knockdown (si-control vs si-TET3) and overexpression (ov-control vs ov-TET3) of TET3 in cell lines were verified with RT-qPCR (A) and Western blot (B).

Supplementary Figure S4


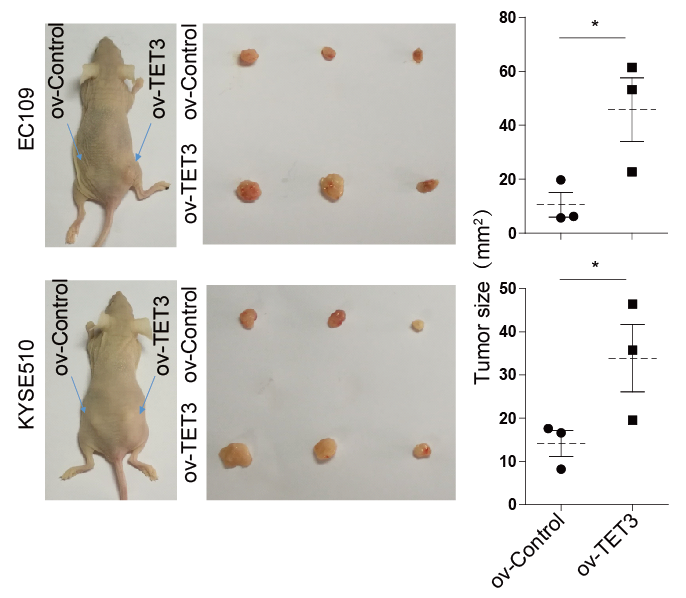


**Supplementary Figure S4** Tumor xenograft was applied to assess the proliferation ability of ESCC cells influenced by TET3 expression. ov-Control group was implanted into the left posterior flank and ov-TET3 group was implanted into the right posterior flank of the same mouse. (ns: no significance, *p <0.05, **p <0.01, ***p < 0.001).

Supplementary Figure S5


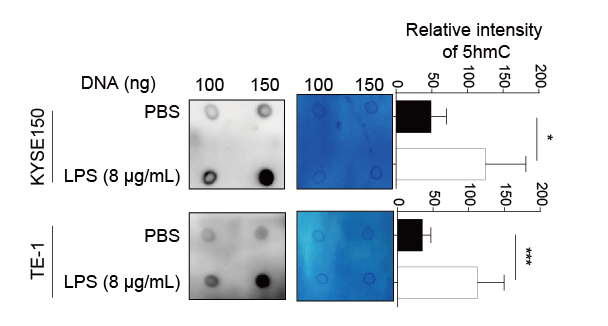


**Supplementary Figure S5** Dot-blot was performed to detect the 5hmC DNA level of ESCC cells with with PBS or LPS stimulation (left panel). The right panel shows the membrane stained with methylene blue as an internal reference.

Supplementary Figure S6


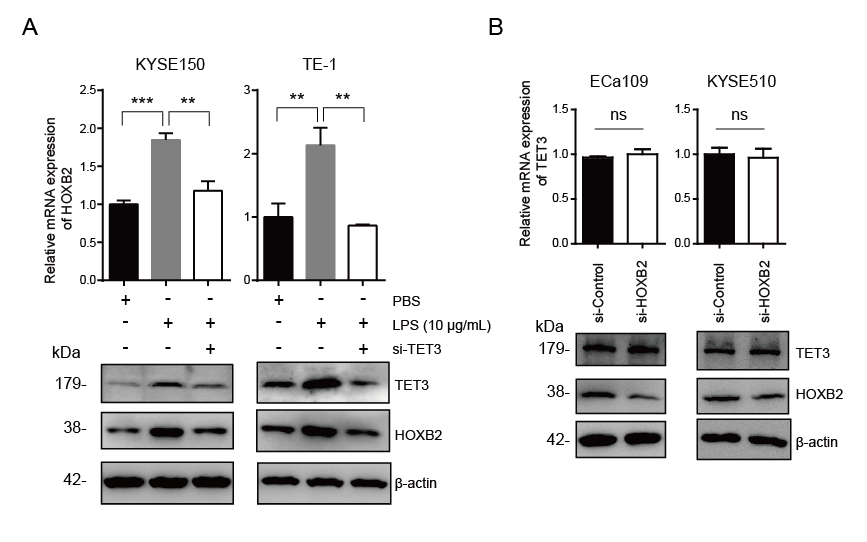


**Supplementary Figure S6** (A) RT-qPCR and Western blot were applied to detect HOXB2 expression upon LPS stimulation and knockdown of TET2. (B) RT-qPCR and Western blot were applied to detect whether knockdown of HOXB2 influenced TET3 expression. (ns: no significance, *p <0.05, **p <0.01, ***p < 0.001).

**Supplementary Figure Legends**

**Supplementary Figure S1** (A) Flow cytometry gating strategies and representative plots. (B) RT-qPCR was performed to detected TET3 mRNA level in ESCC tissues and para-cancerous tissues. (C) RT-qPCR was performed to detected TET3 mRNA level in ESCC cell lines and normal esophageal mucosa epithelium cell line.

**Supplementary Figure S2** Kaplan-Meier product limit estimator was applied to draw overall survival (OS) curves of 299 enrolled ESCC patients, compared according to TET3 expression level, in stage Ⅰ-Ⅱ (A), stage Ⅲ-Ⅳ (B), grade Ⅰ (C) and grade Ⅱ-Ⅲ (D).

**Supplementary Figure S3** The knockdown (si-control vs si-TET3) and overexpression (ov-control vs ov-TET3) of TET3 in cell lines were verified with RT-qPCR (A) and Western blot (B).

**Supplementary Figure S4** Tumor xenograft was applied to assess the proliferation ability of ESCC cells influenced by TET3 expression. ov-Control group was implanted into the left posterior flank and ov-TET3 group was implanted into the right posterior flank of the same mouse. (ns: no significance, *p <0.05, **p <0.01, ***p < 0.001).

**Supplementary Figure S6** (A) RT-qPCR and Western blot were applied to detect HOXB2 expression upon LPS stimulation and knockdown of TET2. (B) RT-qPCR and Western blot were applied to detect whether knockdown of HOXB2 influenced TET3 expression. (ns: no significance, *p <0.05, **p <0.01, ***p < 0.001).

**Supplementary Table S1: Antibodies, inhibitors and other reagents**

| Antibody | Company | Catalog No. |
| --- | --- | --- |
| TLR4 | Affinity | AF7017 |
| TET3 | Genetex | GTX121453 |
| DAPI | Beyotime | C1005 |
| CD133 | Biolegend | 372804 |
| 5hmC | Abcam | ab214728 |
| HOXB2 | Biorbyt | orb214048 |
| ERK | Cell Signaling | 4695S |
| p-ERK | Cell Signaling | 4370S |
| p38 | Cell Signaling | 9212 |
| p-p38 | Cell Signaling | 9215 |
| β-actin | Proteintech | HRP-60008 |
| NF-κB inhibitor (BAY11-7082) | Beyotime | SF0011 |
| MEK inhibitor (SB 202190) | Sigma-Aldrich | S7067 |
| p38 inhibitor (U0126) | Cell Signaling | 9903 |
| Lipopolysaccharides | Sigma-Aldrich | L2880 |

“p” in the given proteins refers to phosphorylated.

**Supplementary Table S2 Sequences for siRNA (5’-3’)**

| **Name** | **siRNA** | **Sequence** |
| --- | --- | --- |
| si-TET3 | forward | CUCUGUCCGAGGUGUCUCAdTdT |
|  | reverse | UGAGACACCUCGGACAGAGdTdT |
| si-HOXB2 | forward | AUCAAGGAGUCGACAUUAA |
|  | reverse | UUAAUGUCGACUCCUUGAU |

**Supplementary Table S3 Primer sequences for PCR (5’-3’)**

| Primers for real time RT-qPCR | | |
| --- | --- | --- |
| **Name** | **Primer** | **Sequence** |
| TET3 | forward | TGCGATTGCGTCGAACA |
|  | reverse | TGCGGATCACCCACTTTG |
| SOX2 | forward | TACAGCATGTCCTACTCGCAG |
|  | reverse | GAGGAAGAGGTAACCACAGGG |
| NANOG | forward | TTTGTGGGCCTGAAGAAAACT |
|  | reverse | AGGGCTGTCCTGAATAAGCAG |
| cMYC | forward | GTCAAAATGGCAGAGATCGAG |
|  | reverse | AGCCTTCATAGTAACAATCACTTC |
| OCT4 | forward | AGAGGATCACCTTGGGGTACA |
|  | reverse | CGAAGCGACAGATGGTGGTC |
| PROM1 | forward | AGTCGGAAACTGGCAGATAGC |
|  | reverse | GGTAGTGTTGTACTGGGCCAAT |
| β-actin | forward | TGACGTGGACATCCGCAAAG |
|  | reverse | CTGGAAGGTGGACAGCGAGG |
|  | | |
| Primers for ChIP-qPCR | | |
| **Name** | **Primer** | **Sequence** |
| cMYC | forward | CGAGGCTGCAGTGAGCTTTG |
|  | reverse | GGAGGCACAGCAGAAGGTGA |
| NANOG | forward | ATTCTCCCGCCTCAGCTTCC |
|  | reverse | CATGGCGAAACCCCCTCTCT |

| PCR primers for the amplification of cMYC and NANOG gene promoters | | |
| --- | --- | --- |
| **Name** | **Primer** | **Sequence** |
| cMYC | HY-KL-01046-p1(KpnI) | AAATTAACCGGGTGTGGT |
|  | HY-KL-01046-p2(Hind III) | CATTGCATTTGTTGGGGGGAGTCAT |
| NANOG | HY-KL-01045-p1(KpnI) | CCTCCCTAGTAGATG |
|  | HY-KL-01045-p2(Hind III) | AATGAAGGCTCTATCACCTTAGACC |
